# The Relationship of Sitting Time and Physical Activity on the Quality of Life in Elderly People

**DOI:** 10.3390/ijerph18041459

**Published:** 2021-02-04

**Authors:** Jung In Choi, Young Hye Cho, Yun Jin Kim, Sang Yeoup Lee, Jeong Gyu Lee, Yu Hyeon Yi, Young Jin Tak, Hye Rim Hwang, Seung Hun Lee, Eun Ju Park, Young In Lee, Young Jin Ra, Su Jin Lee

**Affiliations:** 1 Family Medicine Clinic, Obesity, Metabolism and Nutrition Center and Research Institute of Convergence of Biomedical Science and Technology, Pusan National University Yangsan Hospital, Yangsan 50612, Korea; s1jungin@hanmail.net (J.I.C.); saylee@pusan.ac.kr (S.Y.L.); everblue124@hanmail.net (E.J.P.); ylee23@gmail.com (Y.I.L.); 2 Department of Family Medicine, Pusan National University School of Medicine, Yangsan 50612, Korea; yujkim@pusan.ac.kr (Y.J.K.); eltidine@hanmail.net (J.G.L.); eeugus@pusan.ac.kr (Y.H.Y.); 03141998@naver.com (Y.J.T.); hezera83@naver.com (H.R.H.); b8u5ey@hanmail.net (S.H.L.); yjra@pnuh.co.kr (Y.J.R.); 3 Biomedical Research Institute, Pusan National University Hospital, Busan 49241, Korea; 4 Department of Medical Education, Pusan National University School of Medicine, Yangsan 50612, Korea; 5 Department of Internal Medicine, Division of Infections Disease, Medical Research Institute, Pusan National University School of Medicine, Yangsan 50612, Korea; beauty192@hanmail.net

**Keywords:** sitting time, quality of life, physical activity

## Abstract

Few studies have shown the combined impact of sitting time and physical activity on quality of life in older people. This cross-sectional study, using data from the 2016–2018 Korean National Health and Nutrition Examination Survey, examines the association between sitting time and physical activity and health-related quality of life (HRQoL) in Korean adults aged ≥ 65 years. HRQoL was assessed using the EuroQol-5 Dimension (EQ-5D, three-level version). We divided subjects into groups based on sitting time and physical activity and analyzed the combined association of sitting time and physical activity with HRQoL. The association between longer sitting time (≥8 h) and HRQoL was analyzed using multiple logistic regression. In total, 4276 participants were included. Prolonged sitting time was associated with all of the EQ-5D dimensions: mobility (odds ratio [OR]: 1.43, 95% confidence interval [CI]: 1.22–1.68), self-care (OR: 1.65 [95% CI 1.25–2.17]), usual activities (OR: 2.07 [95% CI 1.69–2.52]), pain/discomfort (OR: 1.57 [95% CI 1.34–1.84]), and anxiety/depression (OR: 1.49 [95% CI 1.17–1.91]). The prolonged sitting time/inactive group had higher ORs for all the EQ-5D dimensions than the low sitting time/active group. Prolonged sitting time was associated with low HRQoL in elderly Korean adults; physical activity could weaken the negative influence of prolonged sitting time on HRQoL.

## 1. Introduction

As life expectancy has gradually increased, it has become increasingly important to improve health-related quality of life (HRQoL) in older people [1]. HRQoL is a multidimensional indicator that covers physical health, psychological status, personal relationships, functional capacity, social support, and life satisfaction [2]. Therefore, HRQoL measures have been used to estimate the health conditions or needs of the older population and to improve public health policies [1,3].

There have been many studies of HRQoL in older people, and quality of life has been found to be influenced by various factors [4]. Physical activity has already been proven to have a positive effect on the quality of life of older people, including physical health, mental health, functional capacity, individual autonomy, and pain [5]. A previous systematic review stated that aerobic training programs had benefits not only for health, such as cardiovascular, metabolic, and cognitive outcomes, but also for improving quality of life in older people (aged ≥70 years) [6].

In contrast, prolonged sitting time is known as a risk factor for adverse health outcomes in older adults [7]. Several studies have found that prolonged sitting time leads to cognitive impairment, mobility limitation, increased risk of mortality, and reduced quality of life in older people [7,8,9,10]. According to the 2015–2016 National Health and Nutrition Examination Survey in the US, 20% of US older adults (aged ≥65 years) sat for at least eight hours a day, and 33% of US older adults sat for six to eight hours [11]. Therefore, it is necessary to make efforts to reduce sitting time in older people because prolonged sitting time has physical and mental health risks [12].

Previous studies have shown that sitting time and physical activity independently affect HRQoL [5,7,13]; however, few studies have examined the combined influence of sitting time and physical activity on HRQoL [14]. This study aims to examine the association between sitting time and HRQoL by controlling for the relevant factors, and to assess the combined effect of sitting time and physical activity on HRQoL in elderly Korean adults.

## 2. Materials and Methods

### 2.1. Subjects

We performed a cross-sectional study using data from the 7th Korean National Health and Nutrition Examination Survey (KNHANES) (2016–2018). The 7th KNHANES was conducted by the Korea Centers for Disease Control and Prevention (KCDC) in South Korea. It was a nationally representative survey of South Koreans that included health interviews, a nutrition survey, and a health examination. Among the 24,269 participants, we excluded the following from the study: those aged <65 years and those without information on sitting times, physical activity, the EQ-5D questionnaire, and/or nutritional status. A total of 3881 participants were enrolled in the study. The present study was approved by the Institutional Review Board of Pusan National University Yangsan Hospital (IRB No. 05-2020-264).

### 2.2. Measures

HRQoL was measured using the EQ-5D-3L questionnaire, which comprised five dimensions: mobility, self-care, usual activity, pain/discomfort, and anxiety/depression. Each dimension was divided into three categories: no problems, moderate problems, and severe problems. The responses for each of the five dimensions were categorized into “having problems” and “having no problems.” The EQ-5D index scores ranged from 1 to −1, where 1 indicates no health problems and −1 indicates a health status no better than death [13,15].

Sitting time was evaluated using the long version of the International Physical Activity Questionnaire (IPAQ) [16], using the following questions: “How many hours do you usually spend sitting or lying down when you are working, at home, with friends, moving to places, reading, writing, watching television, playing games, using the Internet, or listening to music, except for sleeping time?” Responses were divided into a sitting time of <8 h/day and ≥8 h/day according to the median value. 

Physical activity was assessed using the Global Physical Activity Questionnaire presented by the World Health Organization (WHO) [17]. Physical activity (PA) was divided into two categories. Those who engaged in at least 150 min of moderate-intensity PA per week, at least 75 min of vigorous intensity aerobic activity per week, or an equivalent combination of moderate and vigorous intensity activity (1 min of vigorous intensity activity is equal to 2 min of moderate intensity activity) were classified as active; all others were classified as inactive.

Age, sex, household income, educational level, occupation, and marital status were considered as socioeconomic factors. Clinical factors included obesity, current smoking status, alcohol use, depression, and the number of chronic diseases. Obesity was assessed using the body mass index (BMI). BMI was calculated as weight in kilograms divided by the height in meters squared. In terms of smoking status, individuals were classified as either a non-smoker or a current smoker. In terms of alcohol consumption, individuals were classified as those who had consumed more than one drink per month for one year, and non-drinkers. The number of chronic diseases was classified into three categories (0, 1, or ≥2); chronic diseases included high blood pressure, dyslipidemia, stroke, myocardial infarction, angina, arthritis, rheumatoid arthritis, chronic renal failure, asthma, thyroid disease, and chronic hepatitis B.

### 2.3. Statistical Analysis

All statistical analyses were performed using the Statistical Package for Social Sciences (SPSS, Inc., Chicago, USA) version 21.0. To obtain a nationally representative sample, we used a complex sampling design including stratification variables, clusters, and sampling weights.

Baseline characteristics were evaluated using the chi-square test and independent *t*-test. Multiple logistic regression analysis was used to determine the association between prolonged sitting time (≥8 h) and HRQoL. All five dimensions of HRQoL were categorized into “having problems” (including some problems and extreme problems) or “having no problems”, and odds ratios (ORs) and 95% confidence intervals (CIs) were estimated to present the risk of dimension-specific problem status. 

To investigate the combined association of physical activity and sitting time with HRQoL, physical activity was categorized as either inactive (below the level recommended in the WHO guidelines) or active (meeting the level recommended in the WHO guidelines), and sitting time was divided into two categories according to the median value: <8 h/d and ≥8 h/d. Based on these classifications, we created four groups: low sitting time/active, low sitting time/inactive, high sitting time/active, and high sitting time/inactive. Multiple logistic regression was performed by adjusting for variables including age, sex, and comorbidities to assess the risk of poor HRQoL in four groups. Statistical significance was considered for *p*-values < 0.05.

## 3. Results

### 3.1. Baseline Characteristics of the Study Population

Baseline characteristics of the study participants stratified by sitting time are shown in Table 1. Of the 4276 individuals, 43.1% (1842/4276) had a sitting time of <8 h/day and 56.9% (2434/4276) had a sitting time of ≥8 h/day. Approximately 44.2% (1889/4276) of participants were men and 55.8% (2387/4276) were women. The mean age of participants was 72.67 ± 5.03 years. Participants in the prolonged sitting time groups (≥8 h/day) were older, more obese, more inactive, consumed more alcohol, and had lower quality of life scores (*p* < 0.001) than the low sitting time groups (< 8 h/day). However, marital status and current smoking status did not differ between the two sitting time groups. 

### 3.2. Sitting Time and HRQoL

Table 2 shows the estimated ORs for perceived problems in each dimension of the EQ-5D using a multiple logistic regression analysis. After adjusting for age, sex, marital status, educational level, occupation, household income level, physical activity, current smoking status, alcohol intake, chronic disease, and depression, all EQ-5D dimensions of mobility, self-care, usual activity, pain/discomfort, and anxiety/depression were associated with a prolonged sitting time (mobility, OR: 1.43 [95% CI 1.22–1.68]; self-care, OR: 1.65 [95% CI 1.25–2.17]; usual activities, OR: 2.07 [95% CI 1.69–2.52]; pain/discomfort, OR: 1.57 [95% CI 1.34–1.84]; anxiety/depression, OR: 1.49 [95% CI 1.17–1.91]).

### 3.3. Physical Activity and HRQoL

Additionally, multiple logistic regression analysis showed an association between physical activity and HRQoL (Table 3). After adjusting for age and sex, all of the EQ-5D dimensions—mobility, self-care, usual activity, pain/discomfort, and anxiety/depression—were associated with physical inactivity (mobility, OR: 1.62 [95% CI 1.37–1.93]; self-care, OR: 1.92 [95% CI 1.42–2.57]; usual activities, OR: 1.87 [95% CI 1.49–2.33]; pain/discomfort, OR: 1.31 [95% CI 1.09–1.57]; anxiety/depression, OR: 1.30 [95% CI 1.01–1.66]). However, in the model controlling the covariates including age, sex, BMI, sitting time, marital status, educational level, occupation, household income level, current smoking status, alcohol intake, chronic disease, and depression, physical inactivity was associated with the dimensions of mobility (OR: 1.46 [95% 1.22–1.74]), self-care (OR: 1.67 [95% 1.24–2.26]), and usual activity (OR: 1.59 [95% 1.27–2.00]). The association between the dimensions of pain/discomfort and anxiety/depression and physical activity was not statistically significant. 

### 3.4. Combined Association of Sitting Time and Physical Activity with HRQoL

Multiple logistic regression analysis was performed to analyze the combined association of physical activity and sitting time with quality of life (Table 4). The adjusted analysis in the four groups showed that the high sitting time/inactive group had higher ORs for all EQ-5D dimensions than the low sitting time/active group (mobility, OR: 1.90 [95% CI 1.47–2.46]; self-care, OR: 2.14 [95% CI 1.41–3.27]; usual activities, OR: 3.38 [95% CI 2.37–4.82]; pain/discomfort, OR: 1.79 [95% CI 1.38–2.31]; anxiety/depression, OR: 1.69 [95% CI 1.19–2.41]). 

## 4. Discussion

This study examined the association of sitting time and physical activity with HRQoL in older adults. Participants with prolonged sitting time showed significantly worse overall HRQoL, even after adjusting for age, sex, marital status, educational level, occupation, household income level, physical activity, current smoking status, alcohol intake, chronic diseases, and depression. 

Several studies have examined the relationship between sitting time and quality of life in older people [7,18,19]. Garcia-Hermoso et al. found that prolonged sitting time increased the risk of cognitive impairment regardless of the level of physical activity, and moderate-to-vigorous physical activity could attenuate these negative effects [8]. A longitudinal study of community-dwelling older adults in Spain stated that more time spent watching TV was related to a higher risk of depressive and psychological distress symptoms in women [20]. A recent study indicated that knee joint pain, hip joint pain, and low back pain were associated with sitting time (≥7.5 h/day) in older adults [21]. Similarly, a cross-sectional study found that a high level of sitting time (>7 h/day) was related to low back pain, and individuals with a prolonged sitting time with low physical activity had a higher risk of low back pain [22]. Yen et al. showed that prolonged sitting time was significantly associated with limited mobility in older adults. The results of previous studies are consistent with those of the present study in that prolonged sitting time was associated with all of the EQ-5D dimensions (mobility, self-care, usual activity, pain/discomfort, and anxiety/depression). Additionally, we presented the relationship between physical activity and quality of life, independent of sitting time. Both prolonged sitting time and physical activity were independently associated with the EQ-5D dimensions of mobility, self-care, and usual activity. Physical inactivity was not statistically significant in the pain/discomfort and anxiety/depression domains of EQ-5D, independent of sitting time. However, prolonged sitting time was statistically significant in the pain/discomfort and anxiety/depression domains of the EQ-5D, independent of physical activity.

The present study examined the combined impact of sitting time and physical activity on HRQoL by comparing four groups according to their sitting time and physical activity level. Our findings suggest that individuals with a prolonged sitting time and low physical activity levels had a worse HRQoL and that the high sitting time/active group had a lower risk of worse results for the usual activity and pain/discomfort domains of HRQoL than the high sitting time/inactive group. In other words, physical activity could weaken the negative association of prolonged sitting time on the usual activity and pain/discomfort domains of HRQoL. A cross-sectional study conducted in the US on adults (aged ≥20 years) reported that short sedentary time (< 438 min/day) and high moderate-to-vigorous physical activity (≥165 min/week) were associated with better a HRQoL, particularly the general health, physical health, and activity limitation components. However, there was no significant combined association of sedentary behavior and moderate-to-vigorous physical activity with poor mental health [14]. Unlike the results of their study, the present study showed that individuals with a prolonged sitting time and low physical activity had problems associated with anxiety/depression, as well as problems associated with the mobility, self-care, and usual activity domains of quality of life.

Korea has the fastest aging population among the Organization for Economic Cooperation and Development (OECD) countries. By 2050, 71% of the population of Korea is expected to be aged 65 years or older [23]. Prolonged sitting time in the older population has become a major social issue because it causes various problems such as cardiovascular disease, sarcopenia, cognitive impairment, and mortality [8,10]. The reason for prolonged sitting time in the Korean elderly seems to be that more than 90% of them spend their leisure time watching TV or listening to the radio, and they often play sedentary Korean games such as hwatu, janggi, and baduk [18]. It is known that leisure activities in older people are associated with higher life satisfaction. In order to improve the HRQoL of older people, it is essential to develop outdoor leisure programs focusing on physical activities [24]. 

This study has some limitations. First, because of the nature of the cross-sectional study, it is difficult to accurately identify the causal relationship between sitting time and HRQoL. Second, the self-reported data on sitting time and physical activity might be less accurate and reliable than objective measurement data. Third, the EQ-5D has limitations in assessing overall HRQoL. Several studies have suggested that the Short Form 6 Dimensions (SF-6D) is more useful and valid than EQ-5D [25,26]. However, the validity and reliability of the EQ-5D has been proven in previous studies, and it has been widely used as a simple method for evaluating quality of life [27,28,29]. Despite these limitations, the strengths of this study were that a nationally representative sample from South Korea was used and potential confounding factors were adjusted for. Moreover, few studies have examined the combined association of sitting time and physical activity with HRQoL in older people.

## 5. Conclusions

This cross-sectional study using representative data from South Korea suggests that prolonged sitting time and low physical activity are associated with poor general HRQoL in elderly people. Social support as well as interventions that reduce sitting time and encourage physical activity are needed to improve HRQoL in elderly people.

## Figures and Tables

**Table 1:** Baseline characteristics stratified by sitting time (*n* = 4276).

	Sitting Time		
	<8 h (*n* = 1842)	≥8 h (*n* = 2434)	*p* Value ^*^
**Sex**			
Male	876 (47.9)	1013 (40.2)	<0.001
Female	966 (52.1)	1421 (59.8)	
**Age category, mean (SE)**	71.68 (0.14)	73.49 (0.14)	<0.001
65–69	731 (41.0)	701 (29.0)	
70–74	548 (27.4)	632 (24.3)	<0.001
75–79	373 (21.1)	601 (25.1)	
80–84	190 (10.5)	500 (21.7)	
**Obesity**			
No (BMI <25 kg/m^2^)	1186 (66.2)	1443 (59.4)	<0.001
Yes (BMI ≥25 kg/m^2^)	650 (33.8)	964 (40.6)	
**Marital status**			
Single	11 (0.4)	21 (0.9)	0.069
Married	1831 (99.6)	2413 (99.1)	
**Household income level**			
Low	790 (43.0)	1204 (49.1)	
Lower middle	551 (28.7)	618 (25.3)	<0.001
Upper middle	309 (17.8)	334 (13.9)	
High	185 (10.4)	266 (11.7)	
**Educational levels**			
Elementary school	1023 (54.1)	1423 (58.5)	
Middle school	304 (17.2)	340 (13.8)	0.011
High school	326 (18.5)	411 (16.4)	
College or higher	181 (10.2)	254 (11.3)	
**Occupation**			
Office work	63 (3.6)	94 (4.1)	
Sales and Service	119 (6.3)	98 (4.1)	
Agriculture, forestry, and fishery	235 (10.6)	149 (5.3)	<0.001
Machine fitting and simple labor	335 (18.8)	352 (14.3)	
Unemployed, housewife, or student	1083 (60.7)	1736 (72.2)	
**Physical activity**			
Inactive	1135 (60.5)	1801 (74.3)	<0.001
Active	707 (39.5)	633 (25.7)	
**Current smoking status**			
No	1654 (89.9)	2213 (91.6)	0.098
Yes	181 (10.1)	214 (8.4)	
**Alcohol use**			
No	1115 (60.5)	1637 (66.9)	<0.001
Yes	721 (39.5)	789 (33.1)	
**Number of chronic diseases ^a^**			
0	972 (54.1)	1087 (44.6)	
1	299 (16.4)	417 (17.1)	<0.001
≥2	570 (29.5)	930 (38.3)	
**EQ-5D index, mean(SE)**	0.91 (0.003)	0.86 (0.004)	<0.001
**EQ-5D dimensions ^b^**			
Mobility ^c^	541 (28.0)	1053 (42.8)	<0.001
Self-care ^c^	116 (5.7)	325 (12.9)	<0.001
Usual activities ^c^	220 (10.9)	627 (25.0)	<0.001
Pain/discomfort ^c^	554 (28.8)	1048 (42.4)	<0.001
Anxiety/depression ^c^	212 (10.3)	412 (16.0)	<0.001

Data are expressed as numbers (standard error; SE) or number (%); columns total 100%. * *p*-values were obtained from two-sample *t*-tests or chi-square tests. ^a^ Number of chronic diseases including cancer, stroke, myocardial infarction, angina, arthritis, rheumatoid arthritis, asthma, thyroid gland disorder, chronic renal failure, and chronic hepatitis B. ^b^ EQ-5D-3L includes five dimensions: mobility, self-care, usual activity, pain/discomfort, and anxiety/depression. ^c^ Each dimension was categorized as “having problems” or “having no problems,” and subjects reported problems related to their health-related quality of life. BMI, body mass index; EQ-5D, EuroQol-5 dimension.

**Table 2:** Association between sitting time and each EQ-5D dimension.

	Mobility	Self-care	Usual Activity	Pain/Discomfort	Anxiety/ Depression
Model 1					
ST < 8 h	1	1	1	1	1
ST ≥ 8 h	1.63 (1.39–1.91) *	1.97 (1.50–2.59) *	2.32 (1.90–2.83) *	1.68 (1.44–1.95) *	1.60 (1.27–2.00) *
Model 2					
ST < 8 h	1	1	1	1	1
ST ≥ 8 h	1.43 (1.22–1.68) *	1.65 (1.25–2.17) *	2.07 (1.69–2.52) *	1.57 (1.34–1.84) *	1.49 (1.17–1.91) ^†^

Values are presented as odds ratio (95% confidence interval). * *p* value < 0.001; † *p* value < 0.005. Model 1 was adjusted for age and sex; Model 2 was adjusted for age, sex, BMI, marital status, educational level, occupation, household income level, physical activity, current smoking status, alcohol intake, chronic disease, and depression. EQ-5D, EuroQol-5 dimension; OR, odds ratio; CI, confidence interval.

**Table 3:** Association between physical activity and each EQ-5D dimension.

	Mobility	Self-Care	Usual Activity	Pain/Discomfort	Anxiety/Depression
Model 1					
Active	1	1	1	1	1
Inactive	1.62 (1.37–1.93) *	1.91 (1.42–2.57) *	1.87 (1.49–2.33) *	1.31 (1.09–1.57) ^†^	1.30 (1.01–1.66) ‡
Model 2					
Active	1	1	1	1	1
Inactive	1.46 (1.22–1.74) *	1.67 (1.24–2.26) ^†^	1.59 (1.27–2.00) *	1.17 (0.97–1.42)	1.18 (0.91–1.54)

Values are presented as odds ratio (95% confidence interval). * *p* value < 0.001; † *p* value < 0.005; ‡ *p* value < 0.05. Model 1 was adjusted for age and sex; Model 2 was adjusted for age, sex, BMI, sitting time, marital status, educational level, occupation, household income level, current smoking status, alcohol intake, chronic disease, and depression. EQ-5D, EuroQol-5 dimension; ST, sitting time.

**Table 4:** Combined association of sitting time and physical activity with each EQ-5D dimension.

	Mobility	Self-Care	Usual Activity	Pain/Discomfort	Anxiety/ Depression
Low ST & Active (*n* = 707)	1	1	1	1	1
Low ST & Inactive (*n* = 1135)	1.19 (0.90–1.57)	1.10 (0.68–1.78)	1.67 (1.14–2.43) *	1.10 (0.82–1.46)	1.08 (0.75–1.56)
High ST & Active (*n* = 633)	1.10 (0.81–1.51)	0.99 (0.57–1.73)	2.18 (1.46–3.25) *	1.45 (1.08–1.94) *	1.34 (0.91–1.98)
High ST & Inactive (*n* = 1801)	1.90 (1.47–2.46) *	2.14 (1.41–3.27) *	3.38 (2.37–4.82) *	1.79 (1.38–2.31) *	1.69 (1.19–2.41) *

Values are presented as odds ratio (95% confidence interval); * *p* value < 0.001; Adjusted for age, sex, BMI, marital status, educational level, occupation, household income level, current smoking status, alcohol intake, chronic disease, and depression; Low ST: sitting time <8 h/day; High ST: sitting time ≥8 h/day; EQ-5D, EuroQol-5 dimension; ST, sitting time.

## Data Availability

The data presented in this study are openly available in the Korea Centers for Disease Control and Prevention database (URLs: https://knhanes.cdc.go.kr/knhanes/sub03/sub03_02_02.do) for researchers.
